# Differences in Sleep Patterns and Mental Health Problems During Different Periods of COVID-19 Outbreak Among Community-Dwelling Older Men in Hong Kong

**DOI:** 10.3389/ijph.2022.1604363

**Published:** 2022-04-01

**Authors:** Gengze Liao, Bixia Huang, Priscilla Ming Lee, Shi Zhao, Chi Kuen Chan, Lai-Bun Tai, Chun Yuk Jason Tsang, Chi Chiu Leung, Mei-Po Kwan, Lap Ah Tse

**Affiliations:** ^1^ JC School of Public Health and Primary Care, The Chinese University of Hong Kong, Hong Kong, Hong Kong SAR, China; ^2^ Pneumoconiosis Clinic, Department of Health, Hong Kong, Hong Kong SAR, China; ^3^ Pneumoconiosis Mutual Aid Association, Hong Kong, Hong Kong SAR, China; ^4^ Stanley Ho Centre for Emerging Infectious Diseases, The Chinese University of Hong Kong, Hong Kong, Hong Kong SAR, China; ^5^ Department of Geography and Resource Management, Wong Foo Yuan Building, The Chinese University of Hong Kong, Hong Kong SAR, China; ^6^ Institute of Space and Earth Information Science, Fok Ying Tung Remote Sensing Science Building, The Chinese University of Hong Kong, Hong Kong SAR, China

**Keywords:** anxiety, COVID-19, depression, sleep, additive interaction

## Abstract

**Objectives:** To determine the association of sleep with mental health among Hong Kong community-dwelling older men in the context of the COVID-19 pandemic.

**Methods:** This additional analysis was derived from the community-dwelling men aged >60 recruited during three COVID-19 outbreaks (i.e., pre-outbreak, between the second and third wave, and during the third wave) in Hong Kong from July 2019 to September 2020. Sleep and mental health were measured by Pittsburgh Sleep Quality Index questionnaire and Hospital Anxiety and Depression Scale, respectively. Multivariate logistic regression models were performed for the associations between sleep and mental health after considering the outbreaks’ impact.

**Results:** Subjects enrolled between the second and third wave tended to have better sleep but worse mental health. Positive associations between poor sleep and depression (AOR = 3.27, 95% CI: 1.60–7.03) and anxiety (AOR = 2.40, 95% CI: 1.07–5.76) were observed. The period “between second and third wave” was positively associated with depression (AOR = 2.65, 95% CI: 1.22–5.83), showing an additive interaction with poor sleep.

**Conclusion:** The positive association between poor sleep and depression was aggravated by the period “between the second and third wave” among community-dwelling older males in Hong Kong.

## Introduction

Coronavirus disease 2019 (COVID-19), since first identified in December 2019, has swept almost every country around the world [1]. Similar to many regions and countries, the COVID-19 cases in Hong Kong also spiked several times since the repeated relaxation and re-imposition of control measures over the past years [2]. The COVID-19 pandemic not only caused direct physiological damage to the patients but also indirectly posed enormous psychological pressure to people experiencing this pandemic. Alterations of daily routines often led to a lack of outdoor activities and inadequate sunlight exposure due to the stringent control measures, which may adversely impact people’s sleep quality, weaken their circadian rhythm, and worsen their mental health issues during the pandemic [3–8], particularly for the vulnerable older population [9]. A systematic review synthesized evidence from 43 studies and concluded that the community population had higher levels of anxiety and depression during the COVID-19 pandemic than those were observed before the pandemic [4]. Also, studies conducted in the general population and health care workers revealed that more subjects suffered from poor sleep quality during the outbreak than they did before the outbreak [6, 7, 10]. Despite evidence on the association between sleep and mental health problems is still inconsistent [11], recent research tends to support a positive association between poor sleep and depression or anxiety [12–14]. Less has been known whether the pandemic of COVID-19 exaggerates the adverse effect of poor sleep on mental health problems. Therefore, this study aimed to investigate the associations of poor sleep with depression and anxiety among Hong Kong community-dwelling older males in the context of the COVID-19 pandemic. Meanwhile, the roles of different periods of the COVID-19 outbreak on these associations were also explored.

## Methods

### Study Design and the Subjects

The data of this study were derived directly from a cross-sectional study that was originally designed to investigate whether workers with silicosis were more prone to poor sleep quality and mild cognitive impairment using community subjects as the reference. Workers with silicosis were recruited from the annual interview activity organized by the Pneumoconiosis Compensation Fund Board but they were not the study subjects of this study. The current study only involved the community subjects who were recruited from July 2019 to September 2020, which covered three periods of COVID-19 outbreak in Hong Kong (i.e., before the outbreak, during the second and third wave, late phase of the third wave) [15]. We conducted additional analysis of these existing data to understand whether community subjects had changes in sleep and mental health problems during different phases of the COVID-19 outbreak and evaluate the potential impact of the outbreak on the association of sleep and mental health problems among Hong Kong community-dwelling males aged 60 or above. The ethics approval of this study was obtained from the Joint Chinese University of Hong Kong-New Territories East Cluster Clinical Research Ethics Committee (CRE no: 2018.626), and all the written informed consent forms were obtained from the participants before the survey was conducted.

All community participants were recruited separately from the communities located in five different areas of Hong Kong (e.g., Kwun Tong, Kowloon City, Tsuen Wan, Sham Shui Po, and Kwai Tsing District), with age-matched in 5-years to the workers with silicosis. All subjects with silicosis and community subjects were aged above 60 years. The first COVID-19 positive case in Hong Kong was detected on January 23, 2020. We recruited the first batch of 106 community-dwelling males aged >60 before the COVID-19 outbreak (i.e., during July 2, 2019 and August 1, 2019). The second batch of 66 participants was recruited from June 23, 2020 and July 09, 2020, which was the period exactly after the end of the second wave and before the third wave of the COVID-19 outbreak. The third round of recruitment enrolled 70 subjects between September 15, 2020 and September 29, 2020, near the end of the third wave of the outbreak in Hong Kong [2]. Overall, a total of 242 community-dwelling older male subjects were included in this study covering three different periods of the COVID-19 outbreak in Hong Kong. We excluded community subjects with physician-diagnosed psychophysiological problems or other medical conditions that caused them unable to complete the questionnaire such as hearing problems.

### Data Collection and Procedures

Participants were interviewed face-to-face by trained interviewers using standardized questionnaires containing information on socio-demographic characteristics, the level of leisure and physical activity, time of staying at home, dietary habits, tobacco smoking, alcohol drinking, tea and coffee drinking habits, and daily napping duration. We also invited participants to do the anthropometric assessments by directly measuring their height and weight in light cloth and without shoes following a standard protocol; body mass index (BMI) and percentage body fat were measured by body composition monitor (TANITA Corporation, BC-545N) according to the manufacturer’s protocol.

Physical activity was represented by metabolic equivalents (METS) which were assessed by the validated short interviewer-administrated International Physical Activity Questionnaire [16]. The median of METS was used to recategorize the physical activity levels into low and high levels. Leisure-time activity, such as playing computer or watching TV, was also categorized into the low and high levels according to the median of the total time spent on leisure activity per week as the cutoff point. Habitual tea and coffee drinkers were defined as those drinking tea or coffee at least one cup a week over 5 years; otherwise, they were regarded as non-habitual drinkers. Subjects with BMI ≥28 were considered as having obesity.

### Exposure and Outcome Measurements

#### Subjective Sleeping Quality

The Chinese version of the Pittsburgh Sleep Quality Index (PSQI) questionnaire was used to assess subjects’ sleep quality during the month preceding the interview. The PSQI contains seven sleep components: sleep quality, sleep latency, sleep duration, sleep efficiency, sleep disturbance, use of sleeping medication, and daytime dysfunction. Each component is reflected by a score ranging from 0 to 3, where 3 reflects the negative extreme on the Likert scale. The global score is calculated by adding up the scores of the seven components and ranges from 0 to 21. Participants with a global PSQI score >5 were considered as having poor sleep quality; otherwise, the participant had good sleep quality [17]. Each of the seven PSQI components was equally divided into two categories, <2 or ≥2, and a higher score indicates a more negative sleep characteristic [18].

We further extracted the bedtime and self-reported nocturnal sleep duration to calculate the mid-sleep time (MST): MST = bedtime + nocturnal sleep duration/2. The median MST of overall participants was applied to recategorize the subjects into early or delayed MST, and a delayed MST was defined if the participant’s MST was larger than the median value following a 24-h cycle per day, which indicates a delayed sleep phase.

#### Mental Health Assessment

The Chinese version of the Hospital Anxiety and Depression Scale (HADS) was used to evaluate the states of depression and anxiety of each participant. The HADS is a 14-item self-administrated scale including two 7-item subscales: anxiety and depression. Each item used scores on a 0 to 3 Likert scale, yielding a total score with a range from 0 to 21 for anxiety and depression, respectively [19]. The HADS tool has been proven to demonstrate a sensitivity of >80% and specificity of >90% in the Chinese elderly [20]. Subjects with scores for either sub-scale for anxiety or depression of >7 were defined as having possible depression or anxiety; otherwise, the subject was considered normal mental status [21, 22].

### Statistical Analysis

One-way ANOVA and chi-square test were used to compare differences in the continuous and categorical variables between different periods of the COVID-19 pandemic. Unconditional logistic regression models were performed to calculate the odds ratio (OR) or adjusted OR (AOR), and the corresponding 95% confidence interval (95% CI) for the associations between different sleep characteristics, the period effects of different phases of COVID-19 outbreak and mental health problems. Potential confounders included in different multivariate regression models were age, the level of physical activity and leisure activity, the frequency of having dinner after 10 pm, whether they were habitual coffee drinkers or not, period effects of COVID-19 outbreak, and poor sleep quality. Possible multiplicative and additive interactions between various sleep characteristics and the period effects of COVID-19 were tested by a product term (i.e., multiplicative interaction exists if *p*-value was <0.05) and the relative excess risk due to interaction (RERI) (i.e., additive interaction exists if RERI significantly above zero). The calculation of RERI and its 95% CI were performed according to the approach proposed by Tomas Andersson [23]. All statistical analyses were performed by R statistical software version 3.6.3.

## Results

### Sociodemographic Characteristics and Different Patterns of Sleep and Mental Health Problems Before and During COVID-19

The basic characteristics of the participants recruited in the 3 different periods of the COVID-19 pandemic in Hong Kong (i.e., before, between the second and third wave, and during the late phase of third wave) are shown in Table 1. Compared with subjects recruited before the outbreak, more participants recruited between the second and third wave tended to have early sleep phase (MST) and had a significantly better subjective sleep quality, but they returned to a similar level to that observed before the outbreak during the late phase of the third wave. Similar patterns of other sleep characteristics including daily napping duration were also observed between different periods of the outbreak, despite there was no statistical significance. Contrary to sleep, relatively higher scores of depression and anxiety were found among subjects recruited between the second and third wave and these mental health problems tended to be relieved during the late phase of the third wave and were similar to a level close to that was observed before COVID-19, nevertheless, these differences were not statistically significant. Except that more participants had dinner after 10 pm during the late phase of the third wave of the outbreak and more subjects had coffee drinking habits between the second and third wave of the outbreak, there was no significant difference in other sociodemographic characteristics between the 3 phases of the outbreak.

**TABLE 1:** Distribution of selected characteristics among Hong Kong community-dwelling males aged >60 during different waves of COVID-19 outbreak. Sleep Deprivation, Circadian Disruption and Mild Cognitive Impairment among Patients with Silicosis in Hong Kong, Hong Kong SAR, China, 2018–2021.

Characteristics	Before (N = 106)	Between 2nd and 3rd waves (N = 66)	During 3rd wave (N = 70)	*p-*value
Age				0.517
<73 years	56 (52.8)	36 (54.5)	43 (61.4)	
≥73 years	50 (47.2)	30 (45.5)	27 (38.6)	
Education attainment				0.757
Secondary education or below	91 (85.8)	59 (89.4)	62 (88.6)	
Tertiary education	15 (14.2)	7 (10.6)	8 (11.4)	
Physical activity				0.091
Low	62 (58.5)	46 (69.7)	36 (51.4)	
High	44 (41.5)	20 (30.3)	34 (48.6)	
Leisure-time activity				0.576
Low	54 (50.9)	34 (51.5)	41 (58.6)	
High	52 (49.1)	32 (48.5)	29 (41.4)	
Time of staying at home				0.388
<10 h	11 (10.6)	3 (4.5)	7 (10.0)	
10–16 h	48 (46.2)	26 (39.4)	33 (47.1)	
>16 h	45 (43.3)	37 (56.1)	30 (42.9)	
Having dinner after 10 pm				**0.028**
<1 time/week	93 (87.7)	56 (84.8)	50 (71.4)	
<1 time/day	4 (3.8)	6 (9.1)	12 (17.2)	
≥1 time/day	9 (8.5)	4 (6.1)	8 (11.4)	
Tea drinking habits				0.181
Non-habitual drinkers	31 (31.0)	24 (67.5)	31 (44.9)	
Habitual drinkers	69 (69.0)	40 (62.5)	38 (55.1)	
Coffee drinking habits				**0.042**
Non-habitual drinkers	80 (76.9)	45 (69.2)	61 (87.1)	
Habitual drinkers	24 (23.1)	20 (30.8)	9 (12.9)	
Status of obesity				
Non-obesity	92 (86.8)	53 (80.3)	60 (87.0)	0.445
Obesity	14 (13.2)	13 (19.7)	9 (13.0)	
Percentage body fat				0.833
<21%	41 (40.2)	29 (44.6)	30 (43.5)	
≥21%	61 (59.8)	36 (55.4)	39 (56.5)	
PSQI				
Global score (mean ± SD)	6.9 ± 3.6	6.3 ± 3.6	7.3 ± 4.1	0.269
Score for each component (mean ± SD)				
Subjective sleep quality	1.1 ± 0.7	0.9 ± 0.7	1.3 ± 0.9	**0.010**
Sleep latency	1.2 ± 1.0	1.1 ± 1.1	1.2 ± 1.1	0.566
Sleep duration	1.5 ± 1.0	1.4 ± 1.1	1.6 ± 1.0	0.774
Habitual sleep efficiency	1.0 ± 1.1	0.9 ± 1.2	1.2 ± 1.2	0.369
Sleep disturbances	1.3 ± 0.6	1.2 ± 0.5	1.3 ± 0.6	0.409
Use of sleeping medication	0.2 ± 0.8	0.3 ± 0.9	0.2 ± 0.7	0.809
Daytime dysfunction	0.6 ± 0.8	0.4 ± 0.7	0.5 ± 0.9	0.252
MST (Median)	01:50	01:42	01:53	
Early^a^	48 (46.2)	33 (50.0)	31 (44.3)	0.793
Delayed	56 (53.8)	33 (50.0)	39 (55.7)	
Daily napping duration (hours) (mean ± SD)	0.5 ± 0.7	0.8 ± 2.1	0.4 ± 0.8	0.398
Depression (mean ± SD)	4.3 ± 4.0	5.6 ± 4.3	4.5 ± 3.8	0.126
Anxiety (mean ± SD)	3.1 ± 3.2	3.9 ± 4.0	3.5 ± 3.9	0.339

Abbreviations: 2nd, second; 3rd, third; PSQI, the Pittsburgh Sleep Quality Index questionnaire; MST, mid-sleep time.

The results with statistical significance were indicated by bold font.

a Early MST: < 01:45, using overall participants’ median of MST.

### Associations Between Sleep Characteristics and Mental Health Problems

Tables 2, 3 summarize the ORs and 95% CIs for the relationship between sleep characteristics and depression or anxiety, respectively. As shown in Table 2, participants with overall poor sleep (PQSI score > =5) were positively associated with the presence of depression (AOR = 3.27, 95% CI: 1.60–7.03). Among the components of PSQI, higher scores for subjective sleep quality (AOR = 7.41, 95% CI: 3.52–16.34), sleep latency (AOR = 4.57, 95% CI: 2.29–9.43), sleep disturbances (AOR = 2.19, 95% CI: 1.07–4.45), use of sleeping medication (AOR = 2.70, 95% CI: 1.02–6.95), daytime dysfunction (AOR = 3.56, 95% CI: 1.61–7.82) were significantly associated with depression prevalence.

**TABLE 2:** Associations between sleep characteristics and depression status. Sleep Deprivation, Circadian Disruption and Mild Cognitive Impairment among Patients with Silicosis in Hong Kong, Hong Kong SAR, China, 2018–2021.

Characteristics	Without depression	With depression	OR (95%CI)^a^	AOR (95%CI)^b^	AOR (95%CI)^c^
Overall sleep quality					
Good sleep with PQSI <5	92 (48.17)	14 (27.45)	1.00	1.00	1.00
Poor sleep with PQSI ≥5	99 (51.83)	37 (72.55)	**2.46 (1.27–4.99)**	**2.82 (1.42–5.89)**	**3.27 (1.60–7.03)**
PSQI components					
Subjective sleep quality					
Score <2	154 (80.6)	24 (47.1)	1.00	1.00	1.00
Score ≥2	37 (19.4)	27 (52.9)	**4.69 (2.44–9.14)**	**5.65 (2.83–11.59)**	**7.41 (3.52–16.34)**
Sleep latency					
Score <2	134 (70.2)	21 (41.2)	1.00	1.00	1.00
Score ≥2	57 (29.8)	30 (58.8)	**3.36 (1.78–6.43)**	**3.96 (2.04–7.90)**	**4.57 (2.29–9.43)**
Sleep duration					
Score <2	94 (49.2)	20 (39.2)	1.00	1.00	1.00
Score ≥2	97 (50.8)	31 (60.8)	1.51 (0.81–2.88)	1.69 (0.88–3.31)	1.81 (0.93–3.60)
Habitual sleep efficiency					
Score <2	133 (69.6)	33 (64.7)	1.00	1.00	1.00
Score ≥2	58 (30.4)	18 (35.3)	1.25 (0.64–2.39)	1.32 (0.67–2.56)	1.34 (0.68–2.63)
Sleep disturbances					
Score <2	148 (77.5)	32 (62.7)	1.00	1.00	1.00
Score ≥2	43 (22.5)	19 (37.3)	**2.05 (1.04–3.96)**	**2.10 (1.04–4.18)**	**2.19 (1.07–4.45)**
Use of sleeping medication					
Score <2	178 (91.2)	42 (82.4)	1.00	1.00	1.00
Score ≥2	13 (6.8)	9 (17.6)	**2.96 (1.15–7.34)**	**2.71 (1.02–6.93)**	**2.70 (1.02–6.95)**
Daytime dysfunction					
Score <2	169 (88.5)	36 (70.6)	1.00	1.00	1.00
Score ≥2	22 (11.5)	15 (29.4)	**3.22 (1.50–6.81)**	**3.21 (1.47–6.91)**	**3.56 (1.61–7.82)**
MST					
Early	86 (45.5)	26 (51.0)	1.00	1.00	1.00
Delayed	103 (54.5)	25 (49.0)	0.80 (0.43–1.50)	0.79 (0.41–1.53)	0.82 (0.42–1.60)
Daily napping duration					
≤1 h	159 (83.2)	45 (88.2)	1.00	1.00	1.00
>1 h	32 (16.8)	6 (11.8)	0.66 (0.24–1.59)	0.69 (0.24–1.69)	0.64 (0.22–1.59)

Abbreviations: PSQI, the Pittsburgh Sleep Quality Index questionnaire; MST, mid-sleep time; OR, odds ratio; AOR, adjusted OR; 95%CI, 95% confidence interval.

The results with statistical significance were indicated by bold font.

a Model 1 only adjusted for age.

b Model 2 additionally adjusted for physical activity, leisure-time activity, having dinner after 10 pm, habitual coffee drinkers base on model 1.

c Model 3 additionally adjusted for the period effects of COVID-19, outbreak base on model 2.

**TABLE 3:** Associations between sleep characteristics and anxiety status. Sleep Deprivation, Circadian Disruption and Mild Cognitive Impairment among Patients with Silicosis in Hong Kong, Hong Kong SAR, China, 2018–2021.

Characteristics	Without anxiety	With anxiety	OR (95%CI)^a^	AOR (95%CI)^b^	AOR (95%CI)^c^
Overall sleep quality					
Good sleep with PQSI <5	96 (45.93)	10 (30.30)	1.00	1.00	1.00
Poor sleep with PQSI ≥5	113 (54.07)	23 (69.70)	2.06 (0.95–4.76)	2.18 (0.98–5.13)	**2.40 (1.07–5.76)**
PSQI components					
Subjective sleep quality					
Score <2	161 (77.0)	17 (51.5)	1.00	1.00	1.00
Score ≥2	48 (23.0)	16 (48.5)	**3.31 (1.54–7.16)**	**3.48 (1.58–7.70)**	**3.78 (1.69–8.60)**
Sleep latency					
Score <2	137 (65.6)	18 (54.5)	1.00	1.00	1.00
Score ≥2	72 (34.4)	15 (45.5)	1.63 (0.77–3.44)	1.62 (0.75–3.47)	1.69 (0.77–3.67)
Sleep duration					
Score <2	98 (46.9)	16 (48.5)	1.00	1.00	1.00
Score ≥2	111 (53.1)	17 (51.5)	0.89 (0.42–1.89)	0.94 (0.44–2.03)	0.98 (0.46–2.13)
Habitual sleep efficiency					
Score <2	144 (68.9)	22 (66.7)	1.00	1.00	1.00
Score ≥2	65 (31.1)	11 (33.3)	1.08 (0.48–2.33)	1.09 (0.48–2.38)	1.10 (0.48–2.41)
Sleep disturbances					
Score <2	158 (75.6)	22 (66.7)	1.00	1.00	1.00
Score ≥2	51 (24.4)	11 (33.3)	1.65 (0.72–3.60)	1.61 (0.69–3.61)	1.68 (0.71–3.81)
Use of sleeping medication					
Score <2	195 (93.3)	25 (75.8)	1.00	1.00	1.00
Score ≥2	14 (6.7)	8 (24.2)	**4.34 (1.59–11.27)**	**4.13 (1.47–11.10)**	**4.13 (1.46–11.18)**
Daytime dysfunction					
Score <2	182 (87.1)	23 (69.7)	1.00	1.00	1.00
Score ≥2	27 (12.9)	10 (30.3)	**3.20 (1.32–7.50)**	**3.30 (1.33–7.92)**	**3.46 (1.38–8.43)**
MST					
Early	96 (46.4)	16 (48.5)	1.00	1.00	1.00
Delayed	111 (53.6)	17 (51.5)	0.84 (0.40–1.79)	0.99 (0.46–2.17)	1.00 (0.46–2.21)
Daily napping duration					
≤1 h	176 (84.2)	28 (84.8)	1.00	1.00	1.00
>1 h	33 (15.8)	5 (15.2)	0.94 (0.30–2.44)	0.99 (0.31–2.68)	0.96 (0.30–2.61)

Abbreviations: PSQI, the Pittsburgh Sleep Quality Index questionnaire; MST, mid-sleep time; OR, odds ratio; AOR, adjusted OR; 95%CI, 95% confidence interval.

The results with statistical significance were indicated by bold font.

a Model 1 only adjusted for age.

b Model 2 additionally adjusted for physical activity, leisure-time activity, having dinner after 10 pm, habitual coffee drinkers base on model 1.

c Model 3 additionally adjusted for the period effects of COVID-19, outbreak base on model 2.

A significantly positive association also existed between poor sleep (PQSI ≥ 5) and anxiety (AOR = 2.40, 95% CI: 1.07–5.76) after adjustment of potential confounding factors (Table3). Participants with higher scores in some of the components, such as subjective sleep quality (AOR = 3.78, 95% CI: 1.69–8.60), use of sleeping medication (AOR = 4.13, 95% CI: 1.46–11.18), daytime dysfunction (AOR = 3.46, 95% CI: 1.38–8.43), were associated with the presence of anxiety.

### Period Effects of COVID-19 Outbreak on Mental Health Problems

Using subjects recruited before the COVID-19 outbreak as the reference, those recruited between the second and third wave of the outbreak were positively associated with depression with an AOR of 2.65 (95% CI: 1.22–5.83) (Table 4), whereas there was no significant association with subjects recruited during the late phase of third wave of the outbreak. A similar pattern of period effect of COVID-19 outbreak was also indicated on the presence of anxiety, but the association was attenuated during the second and third wave and lacked statistical significance.

**TABLE 4:** The period effect of COVID-19 outbreak on mental health problems. Sleep Deprivation, Circadian Disruption and Mild Cognitive Impairment among Patients with Silicosis in Hong Kong, Hong Kong SAR, China, 2018–2021.

Characteristics	Without depression	With depression	OR (95% CI)^a^	AOR (95% CI)^b^
Period of COVID-19 outbreak				
Before (N = 106)	88 (83.0)	18 (17.0)	1.00	1.00
Between 2nd and 3rd waves (N = 66)	45 (68.2)	21 (31.8)	**2.28 (1.11–4.76)**	**2.65 (1.22–5.83)**
During 3rd wave (N = 70)	58 (82.9)	12 (17.1)	1.01 (0.44–2.25)	1.15 (0.49–2.67)

**Characteristics**	**Without anxiety**	**With anxiety**	**OR (95% CI)^a^**	**AOR (95% CI)^b^**
Period of COVID-19 outbreak				
Before (N = 106)	95 (89.6)	11 (10.4)	1.00	1.00
Between 2nd and 3rd waves (N = 66)	55 (83.3)	11 (16.7)	1.72 (0.69–4.28)	2.01 (0.77–5.27)
During 3rd wave (N = 70)	59 (84.3)	11 (15.7)	1.55 (0.62–3.85)	1.53 (0.59–3.94)

Abbreviations: 2nd, second; 3rd, third; OR, odds ratio; AOR, adjusted OR; 95% CI, 95% confidence interval.

The results with statistical significance were indicated by bold font.

a Adjusted for age only.

b Adjusted for age, physical activity, leisure-time activity, having dinner after 10 pm, habitual coffee drinkers, poor sleep quality.

### Interaction Between Sleep and Specific Period “Between Second and Third Wave of the Outbreak” on Mental Health Problems

Since we only found a significant association between the period between the second and third wave of COVID-19 outbreak and depression, we further explore the possible interactions between this period of the outbreak and the sleep characteristics on depression. The overall poor sleep (PQSI score ≥5) interacted with the period “between the second and third wave of the outbreak” on an additive scale (RERI = 3.66, 95% CI: 0.75, 11.23) to excessively increase the prevalence of depression (Table 5). Several components of the PSQI were also found to have additive interactions with the period “between the second and third wave of the outbreak” on the risk of depression, for example, subjective sleep quality (RERI = 31.60, 95% CI: 4.94, 146.77), sleep latency (RERI = 8.76, 95% CI: 0.48, 32.66), and sleep disturbances (RERI = 5.51, 95% CI: 1.29, 17.41). However, significant multiplicative interaction only existed between sleep disturbances and this specific period on the increased prevalence of depression (*p* value = 0.027).

**TABLE 5:** Additive and multiplicative interaction assessment between sleep characteristics and the specific period “between second and third wave of COVID-19 outbreak” on the association with depression. Sleep Deprivation, Circadian Disruption and Mild Cognitive Impairment among Patients with Silicosis in Hong Kong, Hong Kong SAR, China, 2018–2021.

Characteristics	RERI (95% CI)	*p* value for multiplicative interaction
Overall poor sleep with PQSI ≥5	**3.66 (0.75, 11.23)**	0.158
PQSI components		
Subjective sleep quality ≥2	**31.60 (4.94, 146.77)**	0.055
Sleep latency ≥2	**8.76 (0.48, 32.66)**	0.452
Sleep duration ≥2	1.94 (−0.90, 7.22)	0.502
Habitual sleep efficiency ≥2	−0.02 (−2.74, 2.79)	0.968
Sleep disturbances ≥2	**5.51 (1.29**, **17.41)**	**0.027**
Use of sleeping medication ≥2	4.19 (−1.52, 22.71)	0.351
Daytime dysfunction ≥2	6.03 (−1.24, 31.57)	0.413
Delayed MST	−1.00 (−4.87, 1.08)	0.481
Daily napping duration >1 h	−1.36 (−4.83, 1.12)	0.482

Abbreviations: MST, mid-sleep time; 95% CI, 95% confidence interval; RERI, relative excess risk due to interaction.

The results with statistical significance were indicated by bold font.

RERI was used to assess additive interaction. Models were adjusted for age, physical activity, leisure-time activity, having dinner after 10 pm, habitual coffee drinkers.

## Discussion

This study makes a unique contribution to the literature in determining the role of different periods of COVID-19 outbreak on the association between poor sleep characteristics and mental health problems, i.e., depression or anxiety, which has never been reported previously. We found that the community-dwelling males aged >60 had a relatively better sleep quality but were more likely to be depressed during the period between the second and third wave of the outbreak. Nevertheless, their sleep quality and mental health status during the late phase of the third wave returned to similar levels to those were observed before the COVID-19 pandemic. A positive association between poor sleep and depression was observed, whereas the association was more prominent during the period “between second and third wave of the outbreak,” showing an extra risk of depression due to the interaction between this specific period and poor sleep compared with the summation of their separate effects, but this additive effect only lasted for a short period and it disappeared in the late phase of the third wave of outbreak.

Since the first positive COVID-19 case was reported, Hong Kong had experienced four waves of the outbreak [2], and this study focused on the first three waves of the outbreak. During each wave, the health authority implemented precautionary measures to halt the spread of the virus, including mandatory mask-wearing in all public areas, work-from-home mandates, and social distancing [24]. Since people aged over 60 years old or with pre-existing comorbidity were associated with an increased rate of case fatality [25, 26], the older population was additionally urged to voluntarily obey the control measures which severely disturbed their daily routines [27]. Although older community dwellers might wish to acquire more information about this novel virus for their health, the lower health literacy level and the inadequate ability to acquire information [28, 29] could yield them obtaining less valuable information than they expected, which therefore may lead them to increased psychological distress particularly in the early phase of pandemic [30]. Our study found that the mental health status, i.e., the level of depression and anxiety, among the community-dwelling males aged >60 had the worse mental health problems for the period between the second and third wave of the outbreak than that was observed before the COVID-19 outbreak or the later phase of the third outbreak. A similar phenomenon was found among the community population in England who had the highest levels of depression and anxiety in the first week of lockdown but recovered rapidly in the next couples of weeks [31]. After adjustment of potential confounding factors, our additional analysis confirmed that this particular period, but not the later period of the outbreak, was associated with an increased likelihood of depression among community-dwelling males aged >60. The different period effects of outbreak on depression or anxiety that mental health problems were worse “between second and third wave of outbreak,” but relieved in the late phase of third wave could be explained by the relatively adequate and authentic information about this disease as well as abundant availability of personal protective equipment, masks in particular, at the late phase of the third wave of the outbreak. On the other hand, the control measures for protecting the older people are mainly voluntary at the community level rather than compulsory in old age homes in Hong Kong, so the voluntary control measures may also release the psychological stress of the community-dwelling people. Meanwhile, adaptation could be easier to develop for voluntary measures in contrast with strict lockdowns as time went on.

Previous studies indicated that older people had decreased sleep quality [32] with more complaints of disrupted sleep [33]. This pandemic, as a novel stressful event, had worsened sleep disturbances of older adults, posing them more vulnerable during this unprecedented time than ever before [34]. In our study, the community-dwelling older men tended to have better sleep quality for the period between second and third wave than that was observed before the outbreak. The trajectory of sleep quality across different outbreak periods in our study was in line with a larger UK study, in which some of the participants with pre-pandemic (clinical) insomnia experienced significant improvement in sleep quality during the pandemic and suggested that the effect of lockdown may not harm sleep behavior [35].

Depression and anxiety are the common causes of neuropsychological dysfunction [36, 37] and suicidal tendencies [38, 39]. Identifying potential risk factors for depression and anxiety and modifying these risk factors could be of benefit to reduce the prevalence of mental health problems and the associated negative effects. We observed that poor sleep quality was positively associated with both depression and anxiety in the context of the COVID-19 pandemic, which was consistent with the results of previous studies [12, 40, 41]. Among the components of the PSQI, we additionally distinguished some common components as the predictors for depression and anxiety, including subjective sleep quality, use of sleeping medication, and daytime dysfunction, while sleep latency and sleep disturbances were only driving the occurrence of depression. The results of our study are different from a study published in 2015, in which depression was related to daytime dysfunction, and anxiety was associated with subjective sleep quality, sleep latency among the elderly [42]; however, these results may not be exactly comparable to ours, given different social context and pandemic background between the two studies. Many uncertainties encountered at the beginning of the COVID-19 outbreak were likely to lead the vulnerable aged people more prone to negative mental health outcomes, which were consistent with a more prominent effect observed during the period between the second and third wave of the outbreak, while the long-term adverse impact tended to be attenuated because of the gradual adaption and resilience of the community participants. Since both poor sleep and the COVID-19 pandemic are the contributing factors for distress [4, 12–14], we did observe that poor sleep quality and the specific period “between the second and third waves of the outbreak” additively interacted to have an extra risk of depression than the summation of their separate effects. Among the three components of the PSQI that interacted with the period “between the second and third waves of the outbreak” on an additive scale, sleep disturbances also interacted with this period on a multiplicative scale. These findings imply that maintaining good sleep, in particular keeping fewer sleep disturbances, may be beneficial to decrease the risk of depression, especially in the early phase of the outbreak of diseases or disasters.

This study has several advantages. The recruitment of the study population for this cross-sectional study uniquely covered three periods of the COVID-19 outbreak in the Hong Kong community that has never been studied previously. Therefore, our study provided the first evidence to the current literature on the role of COVID-19 outbreaks on the mental health outcomes among the older community-dwelling males aged >60. However, several limitations should be addressed. First, the cause and effect between poor sleep and mental health problems of depression or anxiety based on the cross-sectional study design are hard to determine; therefore, the causation relationships between sleep quality and mental health issues should be examined in future studies using a cohort design. Second, it would be interesting to investigate the separate association between sleep characteristics and mental health problems for different periods of the COVID-19 outbreak. However, our small sample prevented us from conducting further stratified analysis. In addition, some of the 95% confidence intervals were wide which could be attributed to the small sample size of our study, chance could not be totally excluded. The associations between sleep, the effect of the pandemic and depression or anxiety also might be underestimated and tended to be null, given this small sample size and the relatively low sensitivity of the Hospital Anxiety and Depression Scale in detecting clinical depression [43]. Third, as we recruited community subjects from community centers, selection bias may be a concern. Also, these community participants may not be totally comparable in terms of sociodemographic and other characteristics as they were not from the same cohort but were recruited during different periods of the outbreak. After adjustment of potential confounding factors, better sleep quality was still be observed during the outbreak period “between the second and third wave” but there was lacking statistical insignificance (see Supplementary Table S1). We compared the age structure of our sample with the general male population in Hong Kong, they are comparable [44]. We also found that 2.5% of our participants reported physician-diagnosed depression or anxiety before the interview (i.e., among the six subjects with depression, two of them with anxiety simultaneously, out of the total 242 participants). Therefore, the influence of these pre-diagnosed subjects on the results of our study should be within a tolerable range. Extrapolation to the female population should be cautious since we only recruited male volunteers and gender difference is one of the factors that have been linked to mental health vulnerability [41]. As the first batch of data collection before the pandemic was conducted in July 2019, it co-occurred with the social unrest period and this may potentially affect the mental health of Hong Kong citizens, especially with the elderly population. Future studies can try to explore the potential interaction between this political event and the COVID-19 pandemic on mental health status, but it is hard to distinguish the independent background mental health level based on the current study design. Nevertheless, the COVID-19 pandemic is new and unexpected. Any study covering the period before and during the pandemic would be valuable for understanding the specific effect of various risk factors during the COVD-19 pandemic, compared to the effects before the pandemic, and can inform a better preparation and a sound future study design for the period before and immediately after the subsequent pandemics.

### Conclusion

Results from this study demonstrated that the positive association between poor sleep and depression was further aggravated by the specific period “between the second and third wave” of COVID-19 outbreak but it only lasted for a short period, indicating a strong self-healing ability of the community people in coping with the COVID-19 pandemic. Nevertheless, as positive associations between poor sleep and mental health problems were consistently observed after considering the influences of the outbreak, promotion of sleep-well programs along with psychological consultation are thus recommended for mitigating mental health problems among the older community population.

## Data Availability

The datasets used and/or analyzed during the current study are available from the corresponding author on reasonable request.

## Abbreviations

COVID-19, Coronavirus disease 2019; OR, odds ratio; AOR, adjusted OR; 95% CI, 95% confidence interval; PSQI, the Pittsburgh Sleep Quality Index questionnaire; MST, mid-sleep time; HADS, the Hospital Anxiety and Depression Scale; RERI, the relative excess risk due to interaction.
