# Supplementary material for: Differences in Sleep Patterns and Mental Health Problems During Different Periods of COVID-19 Outbreak Among Community-Dwelling Older Men in Hong Kong
Source: Int J Public Health. 2022 Apr 1;67:1604363. doi: 10.3389/ijph.2022.1604363 (PMC9011365; doi:10.3389/ijph.2022.1604363)
Supplement: Supplementary file 1 [file Table1.docx]

**Supplementary Table 1: The period effect of COVID-19 outbreak on sleep characteristics.**

**Sleep Deprivation, Circadian Disruption and Mild Cognitive Impairment among Patients with Silicosis in Hong Kong, Hong Kong SAR, China, 2018-2021**

|  | AOR (95% CI) ^a^ | | | | | | | | | |
| --- | --- | --- | --- | --- | --- | --- | --- | --- | --- | --- |
|  | Poor sleep | Subjective sleep quality ≥2 | Sleep latency ≥2 | Sleep duration ≥2 | Habitual sleep efficiency ≥2 | Sleep disturbances ≥2 | Use of sleeping medication ≥2 | Daytime dysfunction ≥2 | Delayed MST | Napping time > 1h |
| Period |  |  |  |  |  |  |  |  |  |  |
| Before (N= 106) | 1.00 | 1.00 | 1.00 | 1.00 | 1.00 | 1.00 | 1.00 | 1.00 | 1.00 | 1.00 |
| Between 2^nd^ and 3^rd^ waves (N= 66) | 0.55 (0.29-1.05) | 0.64 (0.29-1.35) | 0.70 (0.35-1.35) | 0.70 (0.37-1.32) | 0.94 (0.47-1.85) | 0.76 (0.36-1.57) | 1.14 (0.38-3.27) | 0.67 (0.26-1.60) | 0.79 (0.41-1.51) | 1.37 (0.58-3.19) |
| During 3^rd^ wave (N= 70) | 0.74 (0.39-1.41) | 1.61 (0.82-3.16) | 1.20 (0.64-2.26) | 0.78 (0.41-1.48) | 1.15 (0.59-2.22) | 0.82 (0.39-1.67) | 0.96 (0.30-2.88) | 0.92 (0.39-2.11) | 1.03 (0.54-1.98) | 0.98 (0.40-2.34) |

Abbreviations: 2^nd^=second, 3^rd^=third, MST=mid-sleep time, AOR=adjusted OR, 95%CI=95% confidence interval.

^a^ Adjusted for age, physical activity, leisure-time activity, having dinner after 10 pm.
